# Physical activity and environmental enrichment regulate the generation of neural precursors in the adult mouse substantia nigra in a dopamine-dependent manner

**DOI:** 10.1186/1471-2202-13-132

**Published:** 2012-10-31

**Authors:** Philipp Klaissle, Anne Lesemann, Petra Huehnchen, Andreas Hermann, Alexander Storch, Barbara Steiner

**Affiliations:** 1 Department of Neurology, Charité University Medicine Berlin; CCM, Charitéplatz 1, Berlin, 10117, Germany; 2 Department of Neurology; Division of Neurodegenerative Diseases, Dresden University of Technology, Dresden, 01307, Germany; 3 Center for Regenerative Therapies Dresden (CRTD), Dresden, 01307, Germany; 4 German Center for Neurodegenerative Diseases (DZNE), Research Site Dresden, Dresden, 01307, Germany

**Keywords:** Physical activity, Environmental enrichment, Dopamine, NG2, Oligodendrocytes, Substantia nigra

## Abstract

**Background:**

Parkinson’s disease is characterized by a continuous loss of neurons within the substantia nigra (SN) leading to a depletion of dopamine. Within the adult SN as a non-neurogenic region, cells with mainly oligodendrocytic precursor characteristics, expressing the neuro-glial antigen-2 (NG2) are continuously generated. Proliferation of these cells is altered in animal models of Parkinson’s disease (PD). Exercise and environmental enrichment re-increase proliferation of NG2^+^ cells in PD models, however, a possible mechanistic role of dopamine for this increase is not completely understood. NG2^+^ cells can differentiate into oligodendrocytes but also into microglia and neurons as observed *in vitro* suggesting a possible hint for endogenous regenerative capacity of the SN. We investigated the role of dopamine in NG2-generation and differentiation in the adult SN stimulated by physical activity and environmental enrichment.

**Results:**

We used the 1-methyl-4-phenyl-1,2,3,6-tetrahydropyridine (MPTP)-model for dopamine depletion and analysed newborn cells in the SN at different maturation stages and time points depending on voluntary physical activity, enriched environment and levodopa-treatment. We describe an activity- induced increase of new NG2-positive cells and also mature oligodendrocytes in the SN of healthy mice. Running and enriched environment refused to stimulate NG2-generation and oligodendrogenesis in MPTP-mice, an effect which could be reversed by pharmacological levodopa-induced rescue.

**Conclusion:**

We suggest dopamine being a key regulator for activity-induced generation of NG2-cells and oliogodendrocytes in the SN as a potentially relevant mechanism in endogenous nigral cellular plasticity.

## Background

The hallmark of Parkinson’s disease (PD) is the degeneration of dopaminergic neurons in the substantia nigra (SN) [1]. Although the adult brain bears the lifelong capacity to generate neural precursor cells and neurons, regeneration of nigral dopaminergic neurons remains controversially discussed [2-5]. In animal models of PD glial cells with features of oligodendrocytic precursors such as the expression of the neuro-glial antigen 2 (NG2) are continuously generated in the adult SN [5,6]. Voluntary physical activity and environmental enrichment robustly regulate proliferation of NG2^+^ oligodendrocytic precursors in the SN [6]. We previously showed a decreased nigral NG2^+^ cell proliferation following 6-hydroxydopamine (6-OHDA)-induced dopamine depletion in rats. This effect was restored by physical activity and environmental enrichment and correlated with improved motor behavior [6]. If and how these new cells could add to an endogenous restorative pool following neurodegeneration remains unclear. It is known, that lack of dopamine leads to decreased generation of neurons [7-10], however the regulation of NG2^+^ cells and oligodendrocytes in PD brain has so far not been described in detail. Few studies investigated orthotopic neural (mainly glial) regeneration in the adult SN, the major origin of dopaminergic pathways, but refused observing neurogenesis [2,3,5,11,12]. It is known, that activity induced proliferation, differentiation and survival of glial cells is regulated differentially from neuronal cells [13-15]. Especially maturation of NG2^+^ cells is strongly influenced by physical exercise and environmental enrichment but also by pathological processes in various areas of the adult brain [14-18].

We analysed newly generated nigral NG2^+^ cells, oligodendrocytes and Nestin-GFP^+^ neural precursors over time, depending on the presence of dopamine and stimulation by physical activity and environmental enrichment. Oligodendrocyte and NG2^+^ derived factors have been postulated to play a neurotrophic/neuroprotective role for dopaminergic neurons [19,20]. On the other hand, disturbances in nigral oligodendrogenesis seem to be pathognomonic in PD brain [21,22]. As dopamine is involved in activity-induced neuroprotection in PD brain [23-25], we analysed new nigral NG2^+^ cells and mature oligodendrocytes following 1-methyl-4-phenyl-1,2,3,6-tetrahydropyridine (MPTP) lesion at various time points combined with physical activity and enriched environment. To differentiate between the dopamine and activity effect on oligodendrocytic and other neural precursors, we used mice expressing the green fluorescent protein (GFP) under the promoter of the neural precursor marker Nestin and quantified newborn cells by incorporation of the proliferation marker bromodeoxyuridine (BrdU). *In vitro* analyses were performed to study the differentiation potential of precursor isolated from the SN following MPTP-treatment. L-dopa treatment was used to rescue a dopamine deficit in MPTP treated mice.

## Results

### Increased numbers but reduced survival of new nigral cells following levodopa-treatment of MPTP-mice

**Figure 1:**
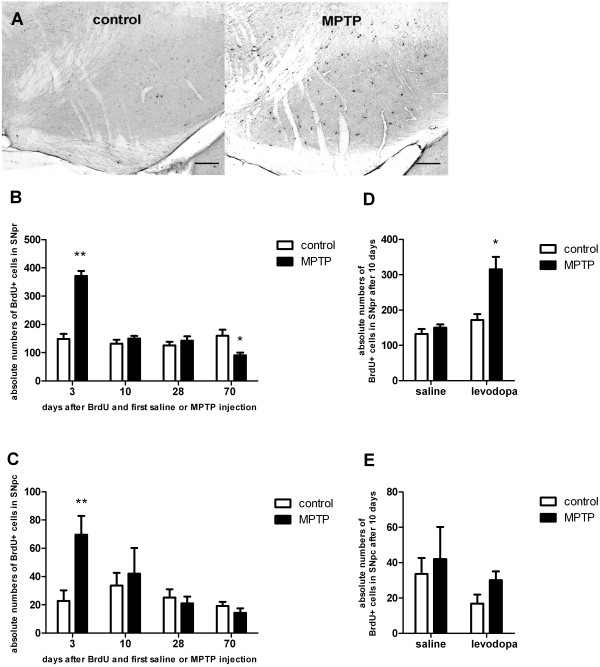
**Histological analysis and quantification of the absolute numbers of BrdU**^**+**^**cells in the substantia nigra pars reticulata (SNpr) and compacta (SNpc) at different time points following BrdU and first saline or MPTP injection.** Data are expressed as mean +/− S.E.M. **A**: BrdU-positive cells in the SN of healthy control animals (control) versus MPTP-treated animals (MPTP). Representative coronal 40μm sections at day 3 after BrdU administration and first saline (control) or MPTP injection are given. Scale bar 100μm. **B**: In short term groups (3 days) MPTP induced a significant increase in the number of BrdU^+^ cells in the SNpr compared to saline-treated controls. In the 70 days-group MPTP caused a significant decrease in the numbers of BrdU+ cells compared to saline-treated controls. *p < 0.05, **p < 0.001 versus corresponding control (n= 5; LSD *post hoc* test). **C**: Only in short term groups (3 days) MPTP induced a significant increase in the number of BrdU^+^ cells compared to saline-treated controls in the SNpc. **p < 0.001 versus corresponding control (n= 5). D: In the SNpr there was a significant interaction effect between MPTP-treatment (MPTP versus control) and levodopa-treatment (L-Dopa versus saline), as levodopa treatment in addition to MPTP-treatment (MPTP + L-Dopa) increased the number of BrdU^+^ cells compared to saline-treated controls (control, saline), MPTP and saline treated mice (MPTP, saline) and levodopa-treated mice. *p < 0.05 versus other groups (n ≥ 5; LSD *post hoc* test). **E**: There were no effects of levodopa-treatment or MPTP on the number of BrdU^+^ cells in the SNpc.

To analyse the effects of MPTP-treatment on generation of newborn cells within the SNpc and SNpr, we quantified BrdU^+^ cells at various time points after MPTP treatment. Three days after BrdU administration, we detected an increase in BrdU^+^ cells in the SN of MPTP-treated mice compared to saline-treated controls in both the SNpc and the SNpr (Figure 1A-C; SNpc: one-way-ANOVA: *p<0001* F_(7;31)_: 3.4*;* SNpr: one-way-ANOVA: *p<0.001* F_(7;31)_: 30.6). At 10 and 28 days after BrdU, no differences in the numbers of BrdU^+^ cells were detected between MPTP-treated and control groups (Figure 1B, C). Long term observations (70d after BrdU and after MPTP), showed a significant decrease in the numbers of BrdU^+^ cells in the MPTP-treated mice compared to controls, indicating a reduced long-term survival of newborn nigral cells in the SN (Figure 1B, C).

After 10 days of levodopa treatment, significant effects of MPTP on the numbers of new nigral cells compared to healthy controls were observed (two-way ANOVA F_(1;16)_: 14.6, *p=0.002*). The same held true for levodopa treatment compared to saline in MPTP-mice (two-way ANOVA F_(1;16)_: 23.4, *p<0.001*; Figure 1D, E). The significant interaction effect (two-way ANOVA F_(1;16)_: 8.8, *p=0.009*) indicates that healthy controls and parkinsonian mice were affected differently by levodopa, as BrdU^+^ cell numbers increased after levodopa treatment in MPTP-mice but not in controls (*p=0.001*). Levodopa-treated MPTP mice also showed an increase in nigral BrdU^+^ cells compared to MPTP-treated mice without substitution of levodopa (*p=0.001*).

### Generation of nigral NG2^+^ cells is increased in levodopa-treated MPTP-mice

**Figure 2:**
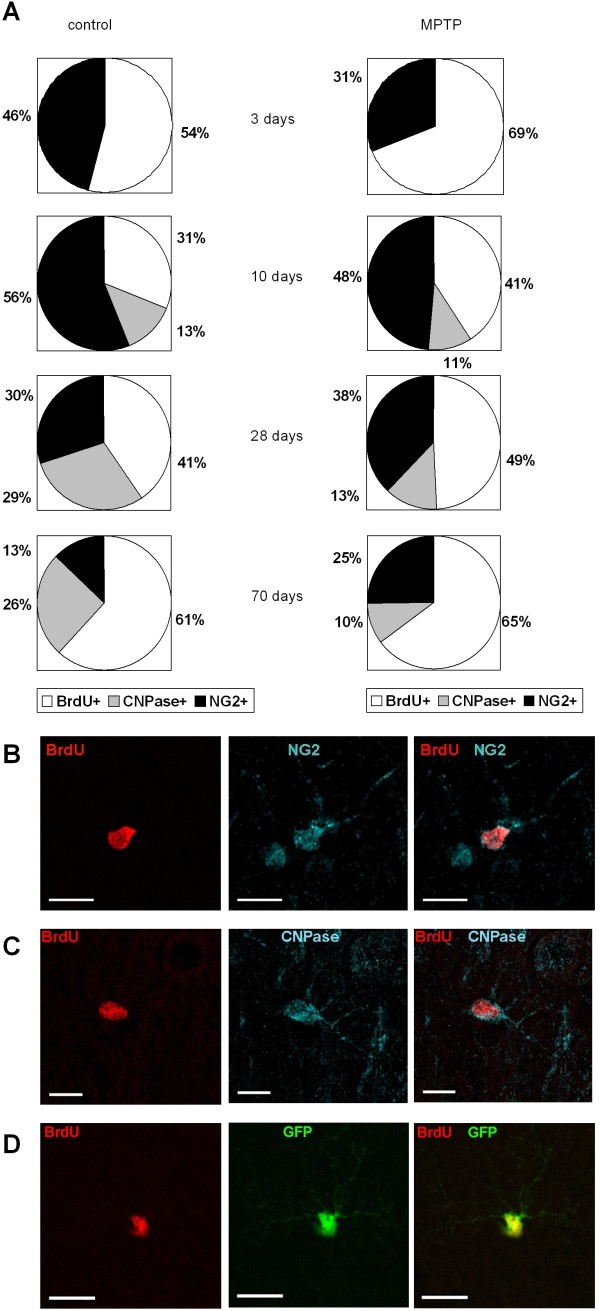
**Co-localization of BrdU-positive cells with different cell markers.A**: Relative numbers of co-localization of NG2-positive cells with BrdU+ cells (NG2+), CNPase- positive cells with BrdU+ cells (CNPase+) and unidentified BrdU+ cells (BrdU+) in saline treated, healthy controls (left panel) and MPTP treated animals (right panel) at different time points. **B**-**D**: Overview of exemplary BrdU+ cells expressing characteristic cell markers in the substantia nigra after 10 days. Left panel shows BrdU (red), central panel shows markers NG2 (blue), CNPase (blue), NesGFP (green), right panel shows overlay. Scale bar 10μm.

**Figure 3:**
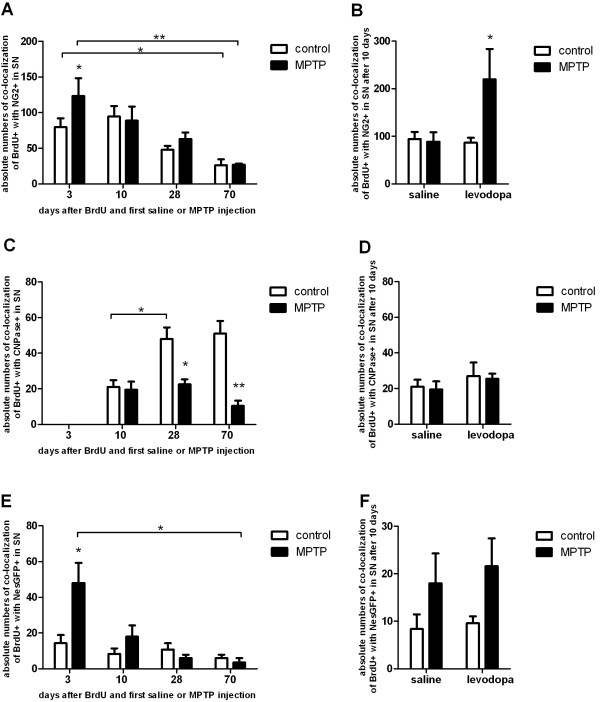
**Histological analysis and quantification of the absolute numbers of co-localization of BrdU-positive cells with NG2**^**+**^**cells, CNPase**^**+**^**mature oligodendrocytes and nestin-GFP**^**+**^**neural precursors in the SN.** Effects of 10 days of levodopa treatment. **A**: In short term groups MPTP induced a significant increase in the number of co-labeled BrdU^+^ and NG2+ in the SN compared to saline-treated controls. At later time points this effect was not detectable. The numbers of co-labeled BrdU^+^ and NG2+ cells decreased over time in controls and MPTP groups. **B**: At day 10 the number of co-labeled BrdU^+^ and NG2+ cells was significantly increased in MPTP mice that were treated with levodopa. **C**: After 3 days no newborn mature oligodendrocytes were detectable. In 28- and 70 day groups MPTP induced a decrease in co-labeled BrdU^+^ and CNPase+ cells compared to saline treated controls where an increase in CNPase+ cells was observed between 10 and 28 days. **D**: At day 10 the number of BrdU^+^-labeled mature oligodendrocytes was not influenced by MPTP or levodopa. There was no significant interaction between levodopa treatment and MPTP-treatment. **E**: In short term groups MPTP induced a significant increase in the number of BrdU^+^ neural precursors (Nes-GFP-positive, Nes-GFP+) in the SN compared to saline-treated controls. In later groups no such effect was detectable. The number of newborn NesGFP+ cells decreased over time in MPTP groups. **F**: At day 10 the number of BrdU^+^ neural precursors was not influenced by MPTP or levodopa. There was no significant interaction between levodopa treatment and MPTP-treatment. *p < 0.05, **p < 0.001 versus corresponding controls, ┌*┐p < 0.05, ┌**┐p < 0.001 versus other groups (n ≥ 5).

At all evaluated time points numerous BrdU^+^ cells expressed NG2. In the three- and ten day groups 46-56% of BrdU-labelled cells of healthy animals were NG2^+^. This percentage decreased over time to 13% after 70 days (Figure 2A). In the acute phase, 3 days after BrdU-injection MPTP led to an increase in the absolute numbers NG2^+^/BrdU^+^ cells in the SN compared to saline-treated controls (one-way ANOVA: *p=0.005*, F_(7;26)_:8.09). At all further time points MPTP had no effect on the numbers of newborn NG2^+^ cells (Figure 2B, 3A).

We observed a significant interaction effect between levodopa and MPTP on the amount of newly generated NG2^+^ cells (2way ANOVA F_(1;13)_ 5.6, *p=0.034*). Specifically levodopa led to an increase of NG2^+^ cells in MPTP- treated mice, but not in healthy animals (Figure 3B).

### Nigral oligodendrogenesis is disturbed by MPTP but remains unchanged by levodopa-rescue

NG2 is described as a marker for oligodendrocytic precursor cells (OPCs) with also a potential to differentiate into neurons or other non-neuronal cells *in vitro* ([19,26-29]. Since a decrease of nigral BrdU^+^ and NG2^+^ cells over time was apparent in all groups we hypothesized that this could be due to maturation into oligodendrocytes. Mature oligodendrocytes were characterized by CNPase-expression. Three days after BrdU-administration no BrdU^+^/CNPase^+^ cells were detected in any group (Figure 2C, 3C). Ten days after BrdU, we found new CNPase^+^ oligodendrocytes in the SN of both MPTP- and saline-treated mice without differences in the numbers of these cells between groups (one-way ANOVA: *p=0.83*, F_(5;18)_:11.5; Figure 3C). An increase in the numbers of BrdU^+^/CNPase^+^ cells over time between 10 and 28 days was detectable in healthy, saline-treated mice in parallel to the decrease of new NG2-positive cells possibly as a sign for oligodendrocytic maturation in the healthy SN (one-way ANOVA: *p=0.001*, F_(5;18)_:11.5; Figure 3C). In parallel, the percentage of CNPase^+^ cells among all BrdU^+^ cells in healthy animals increased from 13% at 10 days to 29% at 28 days and remained at that level at 70 days (26%; Figure 2A). In MPTP treated mice however, there was no difference in BrdU^+^/CNPase^+^ cells at 10 and 28 days (one-way ANOVA: *p=0.67*, F_(5;18)_: 11.5, Figure 3C) and the percentage of CNPase^+^ cells among all BrdU^+^ cells remained stable ranking between 10-13% at all later time points (10, 28, 70 days post MPTP, Figure 2A). Moreover, MPTP induced a significant decrease in the number of BrdU^+^/CNPase^+^ cells compared to healthy controls after 28 days, with continuous decrease of new CNPase^+^ cells at the 70 days time point (one-way ANOVA: *p<0.001*, F_(5;18)_:11.5, Figure 3C). We could not rescue this effect by treatment with levodopa (Figure 3D).

### No influence of dopamine-depletion and levodopa-treatment on nestin-GFP-expressing neural precursor cells

To investigate if the dopaminergic influence also becomes relevant in the stimulation of other neural precursor cell populations in the SN, we quantified Nestin-GFP-expressing cells over time. These cells represent a small population of BrdU+ cells in the SN with decreasing cell numbers over time from 8.4-11.6% on the third day to 1.5-2% after 70 days (Figure 2D, 3E). In the 3 day groups MPTP induced a significant transient increase in the numbers of Nestin-GFP^+^ newborn cells compared to controls as a potential reactive cell response to toxicity. Thus, in the 10 day groups we did not detect any significant differences in numbers of Nestin-GFP^+^/BrdU^+^ cells, this also held true for 28 and 70 days (Figure 3E). Levodopa treatment did not influence the numbers of new Nestin-GFP^+^ cells (two-way ANOVA F_(1;16)_ 0.27, *p=0.61*, Figure 3F).

### The proliferative effect of physical activity and environmental enrichment on nigral cells is suppressed after MPTP-treatment

**Figure 4:**
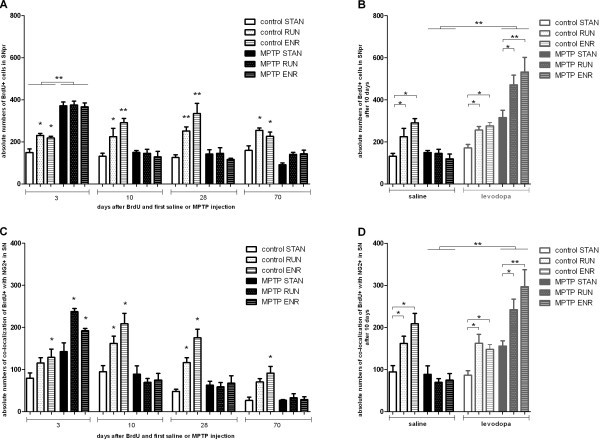
**Effect of physiological stimulation on absolute numbers of BrdU+ cells and doublelabelled BrdU+/NG2+ cells in the SN.A**: In healthy controls RUN and ENR induced a significant increase in the number of BrdU+ cells in the SNpr compared to STAN at all time points. RUN and ENR had no effect in MPTP treated mice at any time point. In short term groups MPTP induced a significant increase in the number of newborn cells in the SN compared to saline-treated controls, independently of physiological stimulation. **B**: At day 10 the number of BrdU+ cells was significantly influenced when MPTP-treated mice additionally received levodopa (p<0.001). There was a significant interaction effect between the three main factors (p=0.001). Specifically, levodopa strengthened the effects of physiological stimulation (RUN and ENR) on BrdU+ cells in MPTP groups compared to controls without levodopa. *p < 0.05, **p < 0.001 versus corresponding STAN. **C**: In healthy controls ENR induced a significant increase in the number of BrdU+/ NG2+ cells in the SN compared to STAN at all time points. RUN induced a significant increase in the number of NG2+ cells in the SN compared to STAN at 10 and 28 days. RUN and ENR significantly increased the number of BrdU+/ NG2+ cells in MPTP treated mice only at the 3d time point. In short term groups MPTP induced a significant increase in the number of BrdU+ /NG2- positive cells in the SN compared to saline-treated controls, independently of physiological stimulation. **D**: At day 10 the number of NG2+ and BrdU+ cells was significantly influenced by levodopa and physiological stimulation. The significant interaction effect between MPTP, levodopa and physiological stimulation shows that levodopa strengthened the effects of physiological stimulation (RUN and ENR) on BrdU+ cells in MPTP groups in contrast to controls without levoopa. *p < 0.05, **p < 0.001 versus corresponding STAN.

We have previously shown the effects of physical activity (RUN) and environmental enrichment (ENR) on nigral cell proliferation in adult rats [6]. In the present study RUN and ENR increased the numbers of BrdU+ cells in the SN of healthy mice at all evaluated timepoints (Figure 4A). RUN and ENR had no effect on the numbers of BrdU^+^ cells in the SN of MPTP-treated animals at any time point, suggesting a role for dopamine in activity-induced nigral cell proliferation (one way ANOVA: 3d: *p=0.9, p=0.84,* 10d: *p=0.87, p=0.46,* 28d: *p=0.92, p=0.38,* 70d: *p=0.08, p=0.05*, F_(23;80)_:19.22, Figure 4A).

### Levodopa rescue re-induces nigral cell generation in MPTP-treated mice

Assuming a key role for dopamine in activity-induced cell proliferation in the SN, we next tested whether the lack of response to physiological stimulation by RUN and ENR MPTP-treated animals could be reversed by administration of levodopa. MPTP-treated mice received levodopa for 10 consecutive days. In these animals, there was a significant main effect of levodopa treatment (three-way ANOVA F_(1;44)_ 93.8, *p<0.001*) and MPTP-treatment (three-way ANOVA F_(1;44)_ 12.63, *p=0.001*), a significant interaction effect between levodopa-treatment and MPTP-treatment (three-way ANOVA F_(1;44)_ 72.1, *p<0.001*) and a significant interaction effect between levodopa, physical stimulation and MPTP (three-way ANOVA F_(2;44)_ 7.88, *p=0.001*) on the numbers of BrdU^+^ cells in the SN (Figure 4B). Thus, the effects of physiological stimulation on the numbers of BrdU^+^ nigral cells could be restored by levodopa. Administration of levodopa to saline-treated controls had no effect on nigral cell proliferation (two-way ANOVA: *p=0.0281* F_(1;22)_ 1.22; Figure 4B).

### Generation of nigral NG2^+^ cells in MPTP-mice induced by RUN and ENR depends on the presence of dopamine

In the next step we analysed the role of activity and levodopa on the numbers of new NG2+ cells in the SN. Except for the short-term exercise group (one-way ANOVA: *p=0.0134*, F_(29;98)_: 18.4) and the 70 days exercise group (one-way ANOVA: *p=0.00*54 F_(29;98)_: 18.4) all healthy controls exposed to RUN and ENR showed an increase in the numbers of BrdU^+^/NG2^+^ cells in the SN compared to controls under standard housing conditions (one-way ANOVA: 3d: *p=0.04*, 10d: *p=0.036, p<0.001,* 28d: *p<0.001,* 70d: *p=0.007*F_(29;98)_: 18.4, Figure 4C).

In MPTP-treated mice, ENR and RUN increased the numbers of newborn NG2^+^ cells only in the short term group (one-way ANOVA: *p<0.001*, F_(29;98)_: 18.4). These differences were no longer apparent at later time points (Figure 4D). When MPTP-treated mice received levodopa for 10 days, there was a significant main effect for levodopa (three-way ANOVA: F_(1;41)_ 34.65, *p<0.001*), a significant interaction effect between levodopa-treatment and MPTP-treatment (three-way ANOVA: F_(1;41)_ 59.1, *p<0.001*) and a significant interaction effect between levodopa, physical stimulation and MPTP (three way ANOVA_ F_(2;21)_ 6.13, *p=0.005*). This indicates that the effect of physiological stimulation on BrdU^+^/NG2^+^ nigral cells in MPTP mice depends on levodopa treatment. In controls, levodopa treatment had no effects (two-way ANOVA: F_(1;20)_ 2.29, *p=0.146*; Figure 4E).

### Disturbed oligodendrogenesis following MPTP is not affected by physiological stimuli or levodopa

The numbers of newly generated CNPase+/BrdU^+^ cells were not changed by physical activity in any groups. In controls after 28 days of exposure to an enriched environment a transient increase in new CNPase^+^ cells could be detected but the differences to the standard housing conditions were not significant over time (one-way ANOVA: 10d: *p=0.04*, *p=0.99*, 28d: *p=0.62, p=0.18*, 70d: *p=0.5, p=0.32,* F_(23;73)_: 6.7). In MPTP mice oligodendrogenesis was not susceptible to physiological stimulation. We neither detected a main effect of physiological stimulation (two-way ANOVA: F_(2;18)_ 0.45, *p=0.64*) nor an interaction effect between levodopa and physiological stimulation on the numbers of CNPase^+^/BrdU^+^ cells (two-way ANOVA: F_(2;18)_ 1.4, *p=0.27*). Levodopa had no effect on CNPase^+^/BrdU^+^ cells (two-way ANOVA: F_(1;18)_ 3.1, *p=0.094*).

### The *in vivo* increase of Nestin-GFP^+^ cells is also reflected in neurosphere-forming cells *in vitro*

**Figure 5:**
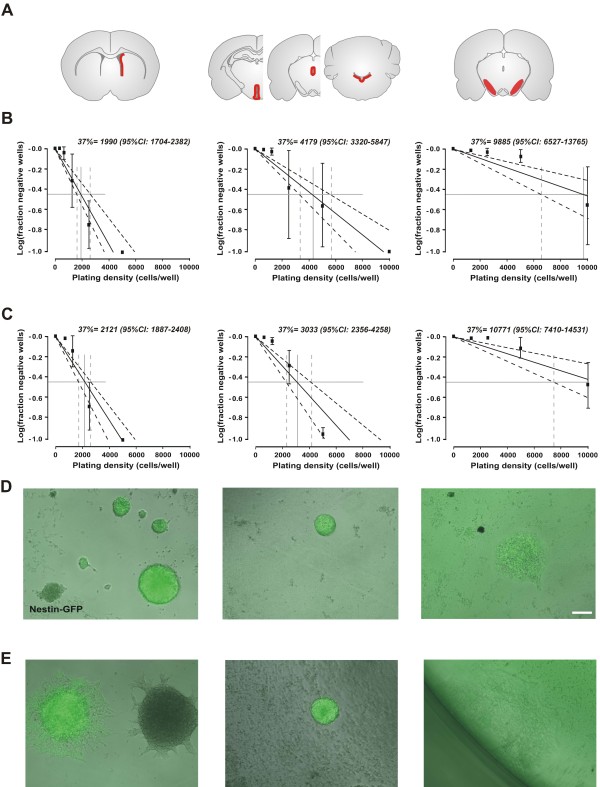
***In vitro *characterization of NPCs derived from microdissected periventricular regions throughout the brain.A**: Schematic brain images showing the regions which were microdissected for NPC culture. **B**: Limiting dilution assay for primary neurospheres from different regions of control mice are shown (n=3). **C**: Limiting dilution assay for primary neurospheres from different regions of MPTP-treated mice are shown (n=3-6). Data are shown as mean±SEM, dotted lines represent 95% confidence interval (95%CI). The intercept of log (37% negative wells) gave the neurosphere-forming frequency. **D**-**E**: Representative neurospheres of NesGFP mice are depicted after one week of expansion of control (D) and MPTP-treated animals (E). Scale bar, 100 μm.

To investigate whether the short term increase of nestin-GFP^+^ cells within the SN after 3 days post MPTP *in vivo* translates into an appearance or increase of neurosphere-forming NPCs *in vitro* as a potential restorative capacity of this cell population, we quantitatively analysed the number of isolable NPCs within the SVZ as well as the SN using the technology introduced by Lefkovits and optimized for the selection of multipotent adult NPCs [3,30,31]. Neurospheres with identical morphology could be detected after 7–10 days *in vitro* independently of the brain’s region of origin. Quantitative colony forming assays revealed an extreme low occurrence of neurosphere-forming cells within the SN (approx. 1 colony-forming cells out of 10,000 isolated cells) with no differences between MPTP-treated and control animals in any brain region (Figure 5). NPCs of the SVZ of both controls and MPTP-treated animals did not show any differences in morphology, nestin-GFP expression (Figure 5) or NPC marker expression (Olig2, Sox2, NG2; data not shown). Of note, proliferation-frequencies of isolated NPCs from the SN were so rare and long-term culture over two weeks and subsequent further characterizations were not possible.

### No newborn neurons, astrocytes or endothelial cells were found in the SN at any time point

We analysed the phenotypic distribution of newly generated BrdU^+^ cells in the SN at all time points following MPTP treatment. Laser confocal microscopy permitted to evaluate the colocalization and quantification of proliferated cells with immature and mature neuronal, glial, and endothelial markers (Nestin-GFP, DCX, NeuN, TH, S100ß, GFAP, NG2, CNPase, Iba1, vWF, CD31). No co-expression of immature (DCX) or mature neuronal markers (NeuN) with BrdU was found at any time point. No proliferated astrocytes (GFAP) or endothelia (anti-vWF) were located in the SN either.

### MPTP induced decrease in TH-immunoreactivity in the SNpc is not reversible by levodopa treatment

**Figure 6:**
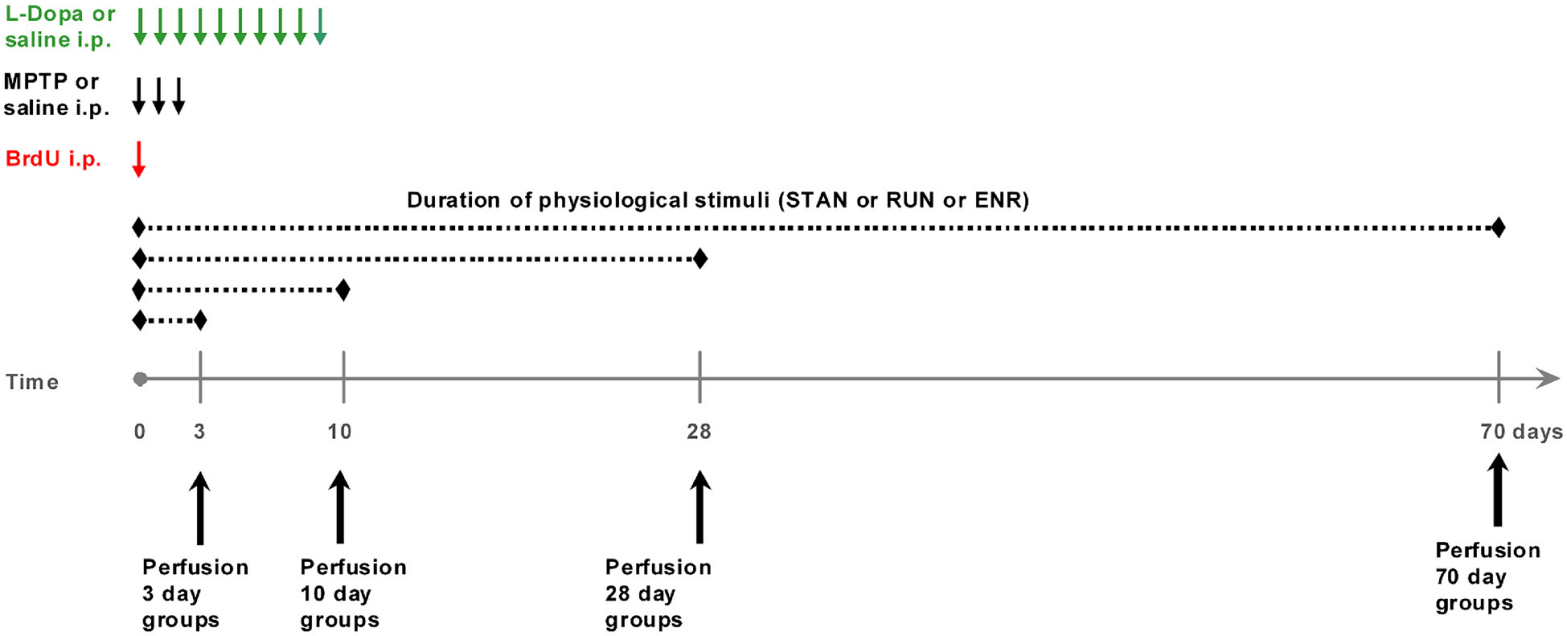
Schematic representation of the experimental design.

**Figure 7:**
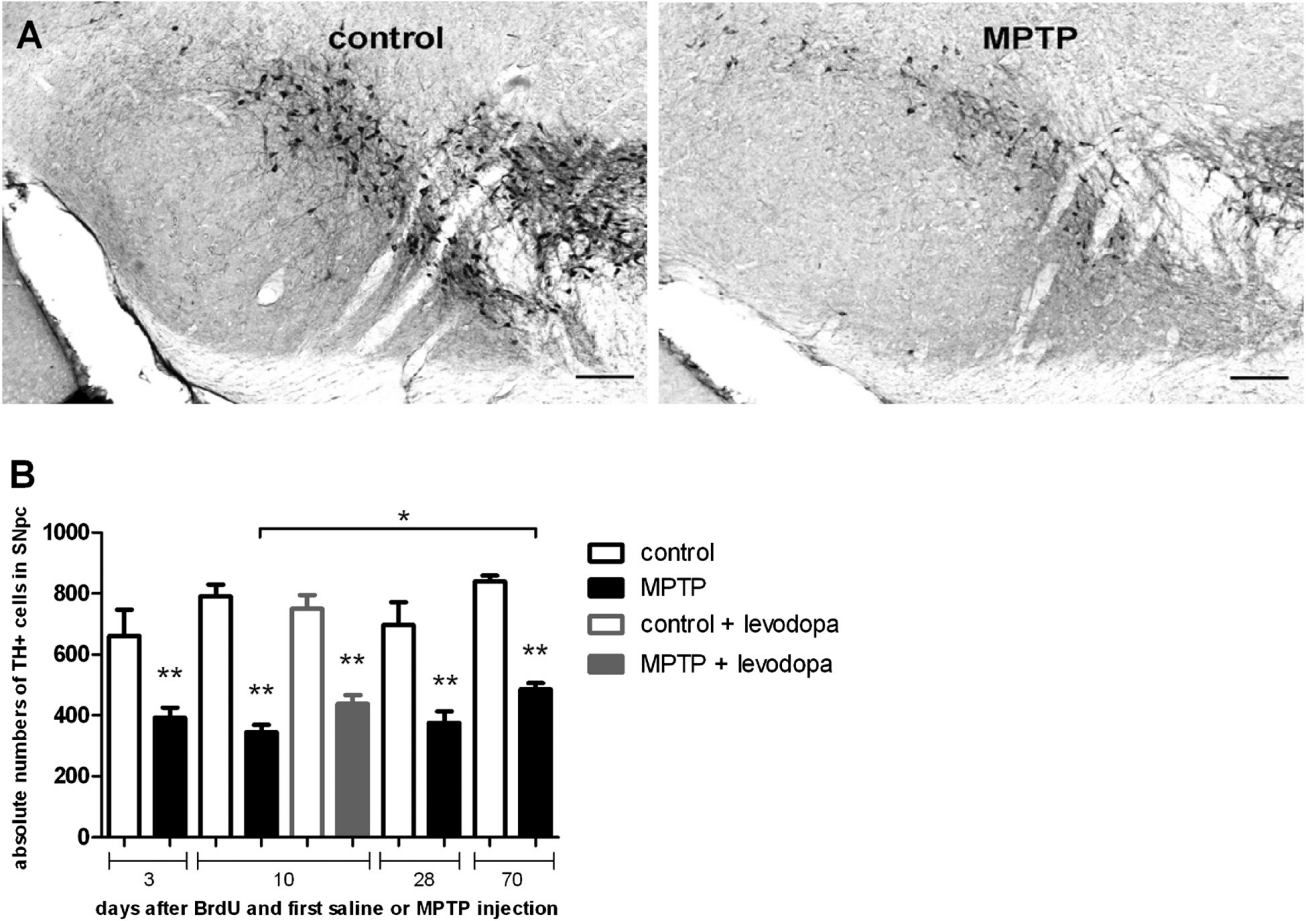
**Histological analysis and quantification of MPTP-induced degeneration of Tyrosinhydroxylase-positive (TH**^**+**^**) dopaminergic neurons in the substantia nigra (SN).A**: TH-expressing dopaminergic neurons in the SN detected by immunohistochemistry. Representative coronal 40μm sections at day 10 after saline (control) or MPTP injection (MPTP) are given. Scale bar 100μm. **B**: MPTP-treatment (MPTP) led to a significant decrease in the numbers of TH^+^ dopaminergic neurons in the SN at all time points compared to controls (control). In MPTP-treated animals there was a recovery in the numbers of TH^+^ neurons after 70 days compared to 10 days. 10 days of levodopa-treatment had no effect on MPTP- treated groups (L-Dopa MPTP) or healthy controls (L-Dopa control). *p < 0.05, **p < 0.001 versus corresponding control (values from LSD *post-hoc* tests).

To assess the effect of MPTP treatment on dopamine depletion in the SN we quantified TH-expressing dopaminergic neurons in the SN at 3, 10, 28 and 70 days after the first MPTP injection (see Figure 6 for experimental design). MPTP-treated animals showed a significant decrease of TH-expressing cell counts compared to saline-treated controls at all analysed time points (Figure 7; one-way ANOVA: *p<0.001* F_(9;42)_: 19.8). After 70 days, TH-immunoreactive cell numbers in MPTP treated mice were increased compared to the cell numbers in MPTP mice after 10 days (one-way ANOVA: *p=0.023;* F_(9;42)_: 19.8). Ten days of levodopa treatment had no effect on the TH-expression, neither in MPTP treated animals, nor in controls (Figure 7; one-way-ANOVA: *p=0.671, p=0.502* ANOVA, F_(9;42)_: 19.8). Physical activity (RUN) and environmental enrichment (ENR) had no effect on TH-positive cell numbers at any time (data not shown).

## Discussion

Although the adult SN lacks the capacity to generate dopaminergic neurons, generation of non-neuronal cells has been robustly shown in the adult nigra [5,6]. The origin and functional role of these cells are still under debate [3]. These cells mainly express the neuro-glial antigen NG2, which marks oligodendrocytic precursors and also a number of cells with the potential to differentiate into neurons or microglia [19,26,27,29]. The main hallmark of PD is dopaminergic neurodegeneration in the SN and the goal for potential restorative therapies in the future might be the replacement of dying tissue by potential neural precursors. We asked, if the generation and differentiation of nigral NG2+ cells depends on dopamine and if and how physical activity and environmental enrichment would influence these cell populations and their potential differentiation. In the first step we quantified all BrdU+ cells in the SNpc and SNpr. As we found that in both areas the numbers of BrdU+ cells were equally significantly increased following MPTP but the absolute numbers of these cells were very low, we did not further differentiate between SNpc and SNpr in the phenotypic analyses of BrdU+ cells. The small numbers of BrdU+ cells in the SN might be due to the usage of only one proliferation marker and its systemic application. Other groups reported the local, intrastrial application of BrdU and consecutively higher numbers of labelled cells [4]. However, intrastriatal application requires a surgical intervention which per se already induces changes in the local microenvironment and might be responsible for changes in the numbers of newborn cells. Thus, the de facto number of BrdU+ cells in the SN might be higher than detected by our methods. The analyses of all BrdU+ cells in the SN over time showed an initial significant increase in the numbers of BrdU+ cells in MPTP-treated mice compared to controls. Within a few days the numbers decrease significantly indicating an initial transitory reactive cell proliferation following the lesion. This hypothesis is supported with the finding, that a large number of these cells were microglia at this time-point and the percentage of microglial cells of all BrdU+ cells did not play a role at any later time point or treatment. One could assume, that after an initial activation these cells either die or migrate away from the lesion site. This migration-hypothesis at the early time point in MPTP-mice would also support the finding, that in healthy control animals the numbers of BrdU+ cells remain stable over a long period of time (70 days in our study) without a significant effect of e.g. aging on the numbers of BrdU+ cells in the SN.

Regarding the phenotypic distribution we found a significant number of the newborn nigral cells co-expressing NG2. These cells were robustly regulated by physiological stimuli in healthy animals as expected from previous work [6,14,15]. In contrast to studies in the 6-OHDA PD rat model, we did not detect an influence of activity on the numbers of NG2^+^ cells of parkinsonian MPTP-treated mice. This might be mainly explained by species and animal model differences. Not till additional treatment with levodopa as dopamine rescue, nigral NG2^+^ cell proliferation in mice with access to physical activity or environmental enrichment was significantly enhanced. These results suggest a function for dopamine in *activity-induced* NG2^+^ cell generation. If these new cells could in the end indeed play a role in neuronal restoration following dopaminergic neurodegeneration remains to be clarified, but their reactive proliferation following MPTP and their vulnerability to physiological stimuli and dopamine suggests a potential function role in the dopamine-depleted brain.

Therefore, in the next step, we were interested if activity had also a dopamine-dependent effect on long-term maturation of NG2^+^ cells. The majority of the NG2^+^ cells in the adult brain *in vivo* are oligodendrocytic precursors [32,33], but NG2^+^ cells in the SN were also described to maturate into microglia or *in vitro* into neurons and secrete neurotrophic factors as a potential endogenous neuroprotective mechanism following neurodegeneration [19,29,34]. The numbers of NG2^+^ cells in our study decreased significantly over time. This decline implicates a physiological differentiation process of NG2^+^ cells into mature oligodendrocytes or other resident cell types in the healthy SN. We thus quantified the numbers of BrdU+ nigral oligodendrocytes by CNPase-expression over time depending on MPTP-treatment and physiological stimulation. In the short-term MPTP- and saline-treated groups, no CNPase^+^ cells were detected indicating the appearance of mature cell populations rather at later time points. The first BrdU^+^/CNPase^+^ cells in the SN were detected 10 days after MPTP or saline in both groups supporting the hypothesis of an ongoing maturation process. In controls the numbers of new CNPase^+^ cells increased over time in line with a continuous decrease of NG2^+^ cell numbers. In contrast, MPTP-treated animals showed unchanged numbers of new CNPase^+^ cells in the SN, despite a continuous decrease of NG2^+^ cells as a possibly sign of a disturbed oligodendrocyte maturation process in the SN following dopamine depletion in line with previous studies showing disturbances in oligodendrocytic homeostasis following MPTP [21]. Another reason for the decrease of NG2^+^ cells in MPTP mice and a lack of increase of CNPase+ cells could be the maturation of NG2^+^ cells in other neural cell types, but also changes due to the aging brain between the earliest and latest time points of our investigations. However, in our model we did not observe any BrdU^+^ cell population increasing over time associated with the NG2^+^ cell decrease.

The numbers of CNPase^+^ oligodendrocytes were not altered by physiological stimulation or levodopa rescue in any group and at any time point. As activity robustly increases functionally relevant neurogenesis in neurogenic regions of the healthy brain [35,36], our data add somewhat to the differentiation between neurogenic and non-neurogenic regions and also show a different regulation of nigral cells following physiological stimuli and pharmacological treatment in line with comparable data from the dentate gyrus [13]. In the SN activity regulated cellular plasticity might occur on precursor cell level, however the stimulus is not sufficient to induce or enhance the further maturation process of these cells. This might be of interest in the context of a suggested neuroprotective role for oligodendrocytes in the lesioned SN most likely by protective factor secretion [37].

The maturation of NG2^+^ cells into microglia with neuroprotective capacities has been discussed recently [19]. Apart from a reactive general glia proliferation 3 days after MPTP, we did not detect any significant changes of Iba1-positive microglia or other glial cells in any group at any time point in this present study. To address if other neural precursors are generated in the SN in a dopamine or activity dependent manner, we applied MPTP to transgenic Nestin-GFP mice and visualised new neural precursors *in vivo*[38,39]. No relevant increase in the numbers of new Nestin-GFP cells was detectable at any time point and treatment paradigm. This is in agreement with the quantitative colony-forming assay results showing no increase of isolatable NPCs following MPTP treatment for 3 days. The acute and transient increase of Nestin-GFP-cells and Iba-1 positive microglia 3 days after MPTP possibly reflects an unspecific reactive post-toxic proliferation [40-42].

After a primary reduction of TH-immunoreactivity, the numbers of immunohistochemically detectable TH^+^ cells in the MPTP-treated mice increased significantly over time [43,44]. The mechanisms underlying this transient degeneration remain widely unclear. No BrdU^+^/TH^+^ neurons have been detected in our study at any time. Thus, the recovery of nigral neurons detected by TH after MPTP administration might be due to a down-regulation of TH-expression on neuronal cells in the SN by MPTP followed by a re-expression of TH over time, rather than by *de facto* degeneration of the neurons or reactive proliferation of new dopaminergic neurons. Both, physiological stimuli and levodopa rescue had no effect on TH-expression in the SN at any time point. One recent report showed forced treadmill-exercise induced increase of the numbers of TH^+^ neurons in the mouse SN [45]. On the other hand, exercise-induced neuroprotection, but no neurogenesis in the SN was reported by others in line with our results [46]. Although one study reported neuronal generation in the SN following MPTP [4], the majority of the studies are in line with our findings of cell proliferation but not neo-neurogenesis in the SN [2,5,6].

Taken together, the activity-induced generation of NG2^+^ precursor cells in the SN critically depends on the presence of dopamine. Based on this data, additional studies will investigate the possible neuro-restorative role of NG2+ nigral cells in PD-brain in dependence of dopamine.

## Conclusions

Physical activity and enriched environment induce generation of newborn cells in the adult mouse SN. The majority of these cells are oligodendrocytic precursors expressing NG2. In the MPTP-model for dopamine depletion the effects of physiological stimuli are suppressed and can be re-activated by levodopa. As NG2^+^ cells have been reported to bear neuroprotective and neuroregenerative capacities in the adult, microenvironmental changes in the SN following activity as a potential future therapeutic strategy in PD should be analysed in future studies based on the present findings.

## Methods

### Animals and housing

All animal experiments were institutionally approved and authorized by the Ethical Committee for Health and Social Care (“Landesamt für Gesundheit und Soziales”) Berlin, Germany. Eight to twelve weeks old female transgenic mice, expressing green fluorescent protein under the promoter of nestin on the genetic background of C57Bl6 mice without any CNS or peripheral pathological phenotype, were randomly assigned to groups of minimum n=5 animals and kept at three different housing conditions with a light/dark cycle of 12 hours and free access to food and water [39]. The housing models consisted of 1. standard housing (‘STAN’) conditions with two mice per cage, 2. cages with voluntary access to a running wheel (‘RUN’) with 2 mice per cage, 3. housing in an ‘enriched environment’ (‘ENR’), with at least 5 mice kept in a cage, 74 x 74 cm, containing changing food locations, obstacles, places to hide, toys, allowing a stimulation of exploratory behavior and social interaction ([35], Figure 6).

### MPTP-model

1-methyl-4-phenyl-1,2,3,6-tetrahydropyridine (MPTP, Sigma-Aldrich) was dissolved in 0.9% NaCl. Half of the mice received 3 intraperitoneal injections of MPTP (20mg/kg body weight) on three consecutive days. Controls were treated with 0.9% NaCl for the same time period (Figure 6).

### BrdU injection

Bromodeoxyuridine (BrdU, Sigma-Aldrich) was dissolved in 0.9% NaCl and filtered. All mice received one intraperitoneal injection of BrdU (50mg/kg body weight) at the first day of MPTP-injection (Figure 6).

### Levodopa treatment

In order to investigate the effect of dopamine substitution in MPTP-treated animals, these mice received daily doses of levodopa + benserazide (L-Dopa, 20mg/kg + 5mg/kg, Ferak Berlin + Sigma- Aldrich) for 10 days starting on day 1 of MPTP or NaCl treatment, respectively. Control mice received 0.9% NaCl under the same conditions (Figure 6).

### Perfusion and tissue preparation

Mice were deeply anesthetized with an overdose of ketamine and killed by transcardial perfusion with 4% paraformaldehyde (PFA) at different time points (day 3, 10, 28 and 70 post MPTP injection). Brains were removed and fixed overnight in PFA at 4°C and then transferred in 20% sucrose for dehydration. Brains were frozen in liquid nitrogen and cut to 40 μm coronal sections using a cryostat (Cryocut 1800, Reichert-Jung) and then stored in cryoprotectant at −20°C until histological analysis.

### Immunohistochemistry

For BrdU staining, DNA was denatured in 2N HCL for 30 min at 37°C. Sections were treated with 0.6% H_2_O_2_ to block endogenous tissue peroxidases. Hereafter sections were washed and incubated with 2N HCL for 30 min at 37°C and rinsed in borate buffer and extensively washed with Tris-buffered saline (TBS). Sections were incubated with the primary antibodies (anti-BrdU, rat, 1:500, Biozol; anti-TH, mouse 1:10 000, Sigma- Aldrich) overnight at 4°C, rinsed in TBS and TBS plus and incubated with the biotinylated secondary antibodies (anti-rat, 1:500, Dianova; anti-mouse, 1:1000, Vector Laboratories) for 2 hours at room temperature. ABC reagent (Vectastain Elite, Vector Laboratories) was applied for 1h at concentration of 9μl/ml. Diaminobenzidine (DAB, Sigma- Aldrich) was used as a chromogen at a concentration of 0.025mg/ml in TBS with 0.01% H_2_O_2_ and 0.04% nickel-II-chloride.

### Immunofluorescence

One-in-six series of nigral sections were triple labelled for immunofluorescence. Sections were incubated with the primary antibodies for 24h at 4°C, washed with TBS and TBS plus and incubated with the secondary antibodies for 4h at room temperature. Sections were then washed, mounted on slides and coverslipped in polyvinyl alcohol diazabicyclo-octane (PVA-DABCO, Sigma- Aldrich) as anti- fading agent. The primary antibodies were applied in the following concentrations: anti-BrdU (rat, 1:500, Biozol), anti-TH (mouse, 1:10,000, Sigma- Aldrich), anti-GFP (goat, 1:1000, APC Biermann Acris; rabbit 1:200, Abcam), anti-Iba1 (rabbit, 1:1000, Wako), anti- NG2 (rabbit, 1:200, Chemicon International), anti-CNPase (rabbit, 1:1000, Abcam), anti- GFAP (guinea pig, 1:500, Advanced ImmunoChemistry), anti-Doublecortin (goat, 1:200, Santa Cruz Biotechnology), anti-S100-beta (mouse, 1:1000, Sigma-Aldrich), anti- activated Caspase3 ( rabbit 1:150, Abcam), anti-von Willebrand Factor (anti- vWF, rabbit, 1:250, Abcam). Secondary antibodies used were: anti-rat Rhodamine X, anti-rabbit FITC, anti-rabbit Cy5, anti-mouse Cy5, anti-guinea pig Cy5, anti-mouse Dylight 405 (all 1:250, Dianova), anti-goat FITC (1:100, Dianova), anti-goat Cy5 (1:500, Dianova).

### Quantification and imaging

One in six sections (240 μm apart) of each brain of all DAB stained animals was counted for BrdU- and TH-positive cells throughout the substantia nigra both, the pars compacta and pars reticulata (SN) bilaterally using a Leica DM RXE microscope. The boundaries of the SN were determined with reference to internal anatomic landmarks using a mouse atlas (The Mouse Brain in Stereotaxic Coordinates, Paxinos 2007) The substantia nigra pars compacta and pars reticulata were delineated at 10x magnification with reference to internal anatomic landmarks. Tyrosine hydroxylase immunostaining was used to approximate the border between the compacta and reticulata area. The SN was outlined in every section using the 10x objective. Actual counting was done using the 40x objective. Resulting numbers were then multiplied by six in order to estimate the total number of stained cells per SN. For immunofluorescence, all BrdU-positive cells in the SN (minimum 18 cells) were analyzed for co-expression of glial, neuronal or vascular markers using a confocal laser scanning microscope (Leica DM 2500) under a 40x- or 63x objective. Absolute numbers and ratios of double- or triple-labelled cells versus BrdU-positive cells are given. All images were taken in sequential scanning mode and further processed in Adobe Photoshop 7.0. Only general contrast adaptations were made and figures were not otherwise manipulated. Exemplary and representative images are presented.

### Statistical analysis

All cell numbers are given as mean with standard errors of the mean (S.E.M). All numerical analysis were performed using SPSS 19. For comparisons one-, two-, and three-way ANOVA was performed, followed by LSD *post hoc* test when appropriate. In one-way ANOVA the factor was ‘groups’, in two-way ANOVA the factors were MPTP treatment (control vs MPTP) and L-dopa treatment (saline vs levodopa) and in three-way ANOVA the factors were MPTP treatment (control vs MPTP), L-dopa treatment (saline vs levodopa) and physiological stimulation (STAN vs RUN vs ENR). Differences were considered statistically significant at p<0.05.

### Neurosphere cultures

Mice were killed by cervical dislocation, their brains removed and placed in ice-cold Hank’s balanced salt solution (HBSS) supplemented with 1% penicillin/strepomycin and 1% glucose (all from Gibco, Tulsa, OK). Whole brains were cut into coronal sections of approximately 500 μm and the SN (defined as remaining tissue of the midbrain/hindbrain region after removal of cerebellum and ventricular zones of third ventricle, Aq, fourth ventricle), as well as the periventricular region of the lateral ventricle (LV, lateral wall) was then aseptically prepared. Expansion of neurospheres was carried out for a maximum of 2 weeks before further characterization [31]. Limiting dilution experiments were done as described by Bull and Bartlett [47]. In brief, primary cells were plated at reducing densities in 96-well plates (BD Biosciences, Australia) with 200 μl per well in Neurobasal® medium supplemented with 1% glutamate, 2% B27 and 1% penicillin/strepomycin containing 20ng/ml EGF (epidermal growth factor) and FGF-2 (fibroblast growth factor 2). Cell suspensions were diluted by serial 1:2 dilutions and cells were plated at 5 to 20,000 cells per well. After 7 days *in vitro*, the fraction of wells negative for neurosphere formation was quantified. These data were then log transformed passing through zero fitted to the data (including the 95% confidence intervals (95%CIs) using the Origin 5.0 software. The intercept of log (37%) gave the frequency of cells capable of proliferating to form a neurosphere [3,30,31].

## Authors’ contribution

PK performed the animal experiments and wrote the manuscript. AL analysed the in vivo material. PH performed the statistical analyses and participated in histological analyses. AH and AS performed the in vitro study and helped to draft the manuscript. BS participated in the in vivo and in vitro analyses, designed the study and wrote the manuscript. All authors read and approved the manuscript.
